# Labia minora elongation: cultural imperialism or mental health concern?

**DOI:** 10.1016/j.eclinm.2023.101948

**Published:** 2023-04-13

**Authors:** Etheldreda Nakimuli Mpungu, Tabitha Aujo, Catherine Abbo, James Okello, Musa Sekikubo, Angela Akol

**Affiliations:** a Department of Psychiatry, College of Health Sciences, Makerere University, Kampala, Uganda; b Department of Mental Health, Gulu University, Gulu, Uganda; c Department of Obstetrics & Gynaecology, College of Health Sciences, Makerere University, Kampala, Uganda; d Ipas African Alliance, Nairobi, Kenya

We have read Kaggwa et al.’s^1^ opinion that appeared in *eClinicalMedicine* on “Labia minora elongation: a neglected form of genital mutilation with mental and sexual health concerns” with keen interest. This is a topic with controversial and opposing views in the literature which we would have loved to see the authors point out so as to have a balanced discussion.^2^^,^^3^ For example, the systematic review referenced by the author points out potential health risks and benefits of LME.^4^ This review concludes that “pain at the beginning of the practice, nuisances related to the use of caustic herbs, and stigmatization in failing to comply with the practice are the principal health risks associated with LME. At the same time, there is evidence that labial elongation may benefit the sexual health and well-being of women.”

We are not aware of any evidence for the claims that LME is a prolonged process that takes months to years of physical and mental suffering. We could not find epidemiological studies comparing any form of distress or mental disorders between those who practice LME and those who do not.

In many African cultures, LME is considered a symbol of femininity and cultural identity.^5^ Therefore, out of respect for those cultures, the calls for laws and regulations to stem the practice must be backed by scientific evidence. There is need for health education on the potential health risks that should be carefully considered when engaging in any form of body modification so that efforts are made to make these practices safe, voluntary and delivered by trained individuals.

## Contributors

All authors contributed equally to this letter.

## Declaration of interests

All authors have no interests to disclose.
